# Supplementary material for: Highs and lows: Genetic susceptibility to daily events
Source: PLoS One. 2020 Aug 13;15(8):e0237001. doi: 10.1371/journal.pone.0237001 (PMC7425846; doi:10.1371/journal.pone.0237001)
Supplement: S1 Appendix — (DOCX) [file pone.0237001.s001.docx]

**English translations of the German stressor/uplift items used in the study**

Items for stressors, uplifts and three further positive affect items were created by the authors. The translations from German into English, presented in this supplemental material, were done by the authors. All response scales were visual analogue scales which ranged from 0 to 100 and indicated agreement with the statements.

| Stressors |  |
| --- | --- |
| *“To what extend did the following statements apply to you during the last hour?”* | |
| Name in data frame | Translation |
| Stressor1_Activity | I would rather do something else |
| Stressor2_Effort | I have to make an effort |
| Stressor3_Unsuccessful | I was not successful in my activities |
| Stressor4_Devalued | Something I have done was devalued by another person |
| Stressor5_Undesirable Task | I attended to my tasks only reluctantly |
| Stressor6_Conflict | I had a conflict with another person |
| Stressor7_ScaryTask | I completed tasks where I could not afford to make any mistakes |

| Uplifts |  |
| --- | --- |
| *“To what extend did the following statements apply to you during the last hour?”* | |
| Name in data frame | Translation |
| Uplift1_Praise | I was praised for something I have done |
| Uplift2_PleasentMoment | I had pleasant moments with other people |
| Uplift3_Successful | I successfully finished tasks |
| Uplift4_Compliment | Somebody gave me a compliment |
|  |  |

The following affect items were added by the authors: “PA5_Satisfied^”^, “PA6_Successful”, and “PA8_Happy”. The remaining items were taken from the German version of the Positive Affect Negative Schedule (Krohne, Egloff, Kohlmann, & Tausch, 1996).

**Factor Analysis for positive and negative affect**

A two-level exploratory factor analysis for ordered-categorical outcomes (cuts at 20, 40, 60, and 80 of the response scale with range 0 to 100) was conducted in Mplus v7.3 to explore the dimensionality of 16 affect items (excluded was the item ‘exhausted’, as it did not show consistent loadings in preliminary analyses). One to three within- and between-person factors were extracted. Eigenvalues suggested extraction of two factors on both levels (Eigenvalues of first four factors were: Within Factor 1: 6.68, Factor 2: 2.66, Factor 3: 1.17, Factor 4: 0.95; Between Factor 1: 7.88, Factor 2: 4.37, Factor 3: 0.92, Factor 4: 0.59), which showed a clear structure of item loadings after oblique Geomin rotation (shown in Table S1). The factors correlated moderately at both levels (Within: r = ­‑.32; Between: r = -.22).

Factor loadings for two-factor solution on within- and between-subject level.

| Affect item | Within | |  | Between | |
| --- | --- | --- | --- | --- | --- |
|  | Factor 1 (negative affect) | Factor 2 (positive affect) |  | Factor 1 (negative affect) | Factor 2 (positive affect) |
| Distressed | .58 | -.31 |  | .80 |  |
| Excited |  | .71 |  |  | .80 |
| Upset | .83 |  |  | .87 |  |
| Strong |  | .74 |  |  | .89 |
| Guilty | .58 |  |  | .82 |  |
| Hostile | .85 |  |  | .77 |  |
| Proud |  | .75 |  |  | .91 |
| Irritable | .79 |  |  | .78 |  |
| Enthusiastic |  | .76 |  |  | .91 |
| Ashamed | .64 |  |  | .85 |  |
| Nervous | .62 |  |  | .83 |  |
| Afraid | .66 |  |  | .89 |  |
| Content | -.37 | .64 |  | -.45 | .67 |
| Successful |  | .76 |  |  | .89 |
| Determined |  | .69 |  |  | .82 |
| Happy | -.36 | .66 |  | -.35 | .72 |

*Note.* Loadings < |.20| omitted.

**Model equations for linear mixed models**

The notation below is adapted from Brincks (2017). Specifically, equations of the main model are shown. The four separate models for positive and negative affect are identically specified. ${mood}_{ijk}$ is the value of the mood index on the *i*th measurement on the *j*th day of person *k*. ${environment}_{PMC}$ are the person mean centered experiences. ${environment}_{GMC}$ are the grand mean centered person average for experiences. Level-1 residuals were modeled via a continuous time autocorrelation structure to account for serial dependence and unequal measurement intervals between adjacent measures.

Genotypes were recoded into Helmert contrasts in accordance with Gunthert and colleagues (2007). In ${HTT}_{contrast1}$, the 5-HTTLPR L/L carriers were assigned the value −2/3; the remaining genotypes were assigned the value 1/3. This contrast reflects the average difference between the L/L and the pooled L/S and S/S carriers on the outcome. In ${HTT}_{contrast2}$, the L/L carriers were assigned the value 0, L/S were assigned the value −1/2, and S/S were assigned the value 1/2. This contrast reflects the average difference between the L/S and S/S.

| Level 1: | ${mood}_{ijk}=\pi_{0jk}+\pi_{1jk}\left( {environment}_{PMC} \right)_{ijk}+e_{ijk}$ | (1) |
| --- | --- | --- |
| Level 2: | $\pi_{0jk}=\beta_{00k}+r_{0jk}$  $\pi_{1jk}=\beta_{10k}$ | (2)  (3) |
| Level 3: | $\beta_{00k}=\gamma_{000}+\gamma_{001}\left( \bar{{environment}_{GMC}} \right)_{k}+\gamma_{002}\left( {HTT}_{contrast1} \right)_{k}+\gamma_{003}\left( {HTT}_{contrast2} \right)_{k}$  $+\gamma_{004}\left( \bar{{environment}_{GMC}} \right)_{k}\times\left( {HTT}_{contrast1} \right)_{k}$  $+\gamma_{005}\left( \bar{{environment}_{GMC}} \right)_{k}\times\left( {HTT}_{contrast2} \right)_{k}+u_{00k}$  $\beta_{10k}=\gamma_{100}$+ $\gamma_{101}\left( {HTT}_{contrast1} \right)_{k}+\gamma_{102}\left( {HTT}_{contrast2} \right)_{k}+u_{10k}$ | (4)  (5) |

**Bootstrapping Procedure**

The confidence intervals for the cross-over point between lines of the genotype groups were estimated via bootstrapping by resampling the 326 participants 10,000 times, using the R packages *boot* and *lmeresampler*. The regression weights of the 5-HTTLPR main effect were divided by the regression weights of the interaction to calculate the intersection $C$ for each repetition, using the equation $C=-\frac{\gamma_{002}}{\gamma_{004}}$ (Widaman et al., 2012). Then, quantiles of the intersections were calculated to get the 95% CIs. Notably, the intersection was calculated between the lines of the L/L-carriers and the pooled S-carriers, because as L/S and S/S carriers did not differ substantially.

**Nonlinear models for negative affect**

Negative affect was recoded into a 5-step categorical variable, consistent with the results of the factor analysis. An ordinal multilevel regression was conducted on categorical negative affect with either uplifts or stressors as the predictor variable. Calculations were done using the function *clmm* of the R-package *ordinal*.

First, models with all the fixed and random components of the linear models were calculated. These models did not converge. Several steps were taken to reach convergence: First, person-means were excluded from the models. Second, stressors and uplifts were divided by 10. This can help when convergence issues are related to a small variance of the random slopes. After applying this procedure, the model using stressors converged. The model using uplifts as predictors only converged when additionally removing the random intercept variance across days. Hence, for the uplift model, a correlated random intercept and random slope across individuals was estimated, but there were no random variances estimated across days (effectively reducing this model to a two-level model).

**References**

Gunthert, K. C., Conner, T. S., Armeli, S., Tennen, H., Covault, J., & Kranzler, H. R. (2007). Serotonin Transporter Gene Polymorphism (5-HTTLPR) and Anxiety Reactivity in Daily Life: A Daily Process Approach to Gene-Environment Interaction. *Psychosomatic Medicine*, *69*(8), 762–768. doi: 10.1097/PSY.0b013e318157ad42

Krohne, H. W., Egloff, B., Kohlmann, C. W., & Tausch, A. (1996). Untersuchungen mit einer deutschen Version der" Positive and Negative Affect Schedule"(PANAS). *Diagnostica-Gottingen*, 42, 139-156.

Widaman, K. F., Helm, J. L., Castro-Schilo, L., Pluess, M., Stallings, M. C., & Belsky, J. (2012). Distinguishing ordinal and disordinal interactions. *Psychological Methods*, *17*(4), 615–622. https://doi.org/10.1037/a0030003
