# Supplementary figures and images for: Highs and lows: Genetic susceptibility to daily events
Source: PLoS One. 2020 Aug 13;15(8):e0237001. doi: 10.1371/journal.pone.0237001 (PMC7425846; doi:10.1371/journal.pone.0237001)

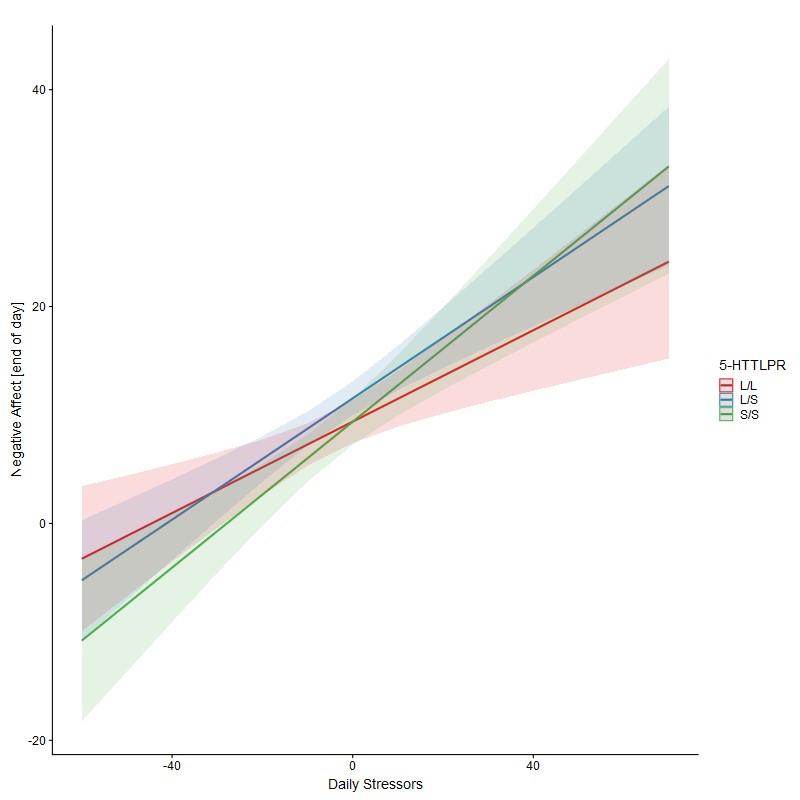

Supplement: S1 Fig — (JPG) [file pone.0237001.s002.jpg]
