# Supplementary material for: Highs and lows: Genetic susceptibility to daily events
Source: PLoS One. 2020 Aug 13;15(8):e0237001. doi: 10.1371/journal.pone.0237001 (PMC7425846; doi:10.1371/journal.pone.0237001)
Supplement: S1 Table — (DOCX) [file pone.0237001.s003.docx]

| Table S1 |  |  |  |  |  |  |  |
| --- | --- | --- | --- | --- | --- | --- | --- |
| Gene–environment interactions predicting affect on the between-person level | | | | | | | |
|  | L/L vs S | | |  | L/S vs S/S | | |
| Predictor | Estimate (*SE*) | 95% confidence  interval | *p* |  | Estimate (*SE*) | 95% confidence  interval | *p* |
| *Positive Affect* |  |  |  |  |  |  |  |
| Stressors | 0.22 (0.23) | [−0.24, 0.68] | .350 |  | 0.30 (0.27) | [−0.24, 0.83] | .276 |
| Uplifts | −0.11 (0.10) | [−0.30, 0.08] | .242 |  | −0.05 (0.11) | [−0.26, 0.16] | .639 |
| *Negative Affect* |  |  |  |  |  |  |  |
| Stressors | 0.15 (0.09) | [−0.03, 0.33] | .106 |  | −0.03 (0.11) | [−0.24, 0.19] | .794 |
| Uplifts | −0.01 (0.07) | [−0.16, 0.13] | .859 |  | 0.13 (0.08) | [−0.03, 0.30] | .118 |
| *Note.* The first three result columns report the contrast of the L/L- against the pooled L/S- and S/S-carriers. A positive sign indicates a more positive slope in the S-groups. The three rightmost columns report the contrast of the L/S against the S/S carriers. Here, a positive sign indicates a more positive slope in the S/S-group, compared to the L/S-group. | | | | | | | |
