# Supplementary material for: Highs and lows: Genetic susceptibility to daily events
Source: PLoS One. 2020 Aug 13;15(8):e0237001. doi: 10.1371/journal.pone.0237001 (PMC7425846; doi:10.1371/journal.pone.0237001)
Supplement: S2 Table — (DOCX) [file pone.0237001.s004.docx]

| Table S2  *Ordinal mixed regression of negative affect on stressors.* | | | |
| --- | --- | --- | --- |
| Predictors | Odds Ratios | *CI* | *p* |
| (Intercept: 1\|2) | 15.07 | 10.87 – 20.91 | **<0.001** |
| (Intercept: 2\|3) | 198.59 | 132.85 – 296.87 | **<0.001** |
| (Intercept: 3\|4) | 1913.51 | 1117.62 – 3276.17 | **<0.001** |
| (Intercept: 4\|5) | 30033.48 | 9661.67 – 93359.65 | **<0.001** |
| Stressors | 2.12 | 1.91 – 2.35 | **<0.001** |
| 5-HTTLPR: L/L vs S | 1.53 | 0.83 – 2.82 | 0.176 |
| 5-HTTLPR: L/S vs S/S | 0.57 | 0.29 – 1.12 | 0.101 |
| Stressors$\times$ 5-HTTLPR: L/L vs S | 1.03 | 0.87 – 1.22 | 0.713 |
| Stressors$\times$ 5-HTTLPR: L/S vs S/S | 1.01 | 0.84 – 1.21 | 0.928 |
| *Note.* 4,905 observations. The standard deviations of random effects were $\sigma_{u_{00k}}$ = 2.10 (person intercepts), $\sigma_{u_{10k}}$ = 0.24 (person slopes), and $\sigma_{r_{00k}}$ = 0.66 (day intercepts). This table was created with the *tab_model* function of the R-package *sjPlot*.  *p*-values ≤ .05 are shown in boldface. | | | |
