# Supplementary material for: Highs and lows: Genetic susceptibility to daily events
Source: PLoS One. 2020 Aug 13;15(8):e0237001. doi: 10.1371/journal.pone.0237001 (PMC7425846; doi:10.1371/journal.pone.0237001)
Supplement: S3 Table — (DOCX) [file pone.0237001.s005.docx]

| Table S3  *Ordinal mixed regression of negative affect on uplifts.* | | | |
| --- | --- | --- | --- |
| Predictors | Odds Ratios | *CI* | *p* |
| (Intercept: 1\|2) | 10.01 | 7.71 – 13.01 | **<0.001** |
| (Intercept: 2\|3) | 89.79 | 65.85 – 122.41 | **<0.001** |
| (Intercept: 3\|4) | 657.39 | 429.47 – 1006.25 | **<0.001** |
| (Intercept: 4\|5) | 8273.61 | 2942.90 – 23260.27 | **<0.001** |
| Uplifts | 0.82 | 0.76 – 0.88 | **<0.001** |
| 5-HTTLPR: L/L vs S | 1.47 | 0.88 – 2.47 | 0.144 |
| 5-HTTLPR: L/S vs S/S | 0.63 | 0.36 – 1.12 | 0.113 |
| Uplifts$\times$ 5-HTTLPR: L/L vs S | 1.01 | 0.90 – 1.14 | 0.864 |
| Uplifts$\times$ 5-HTTLPR: L/S vs S/S | 0.99 | 0.88 – 1.13 | 0.917 |
| *Note.* 4,905 observations. The standard deviations of random effects were $\sigma_{u_{00k}}$ = 1.74 (person intercepts) and $\sigma_{u_{10k}}$ = 0.20 (person slopes). This table was created with the *tab_model* function of the R-package *sjPlot*.  *p*-values ≤ .05 are shown in boldface. | | | |
