# Supplementary material for: Highs and lows: Genetic susceptibility to daily events
Source: PLoS One. 2020 Aug 13;15(8):e0237001. doi: 10.1371/journal.pone.0237001 (PMC7425846; doi:10.1371/journal.pone.0237001)
Supplement: S4 Table — (DOCX) [file pone.0237001.s006.docx]

| Table S4  *Ordinal mixed regression of last daily negative affect measure on average daily stressors.* | | | |
| --- | --- | --- | --- |
| Predictors | Odds Ratios | *CI* | *p* |
| (Intercept: 1\|2) | 13.30 | 8.92 – 19.82 | **<0.001** |
| (Intercept: 2\|3) | 98.72 | 57.83 – 168.51 | **<0.001** |
| (Intercept: 3\|4) | 735.55 | 334.39 – 1617.98 | **<0.001** |
| (Intercept: 4\|5) | 16322.02 | 1754.83 – 151814.67 | **<0.001** |
| Stressors | 1.09 | 1.05 – 1.12 | **<0.001** |
| 5-HTTLPR: L/L vs S | 1.41 | 0.75 – 2.65 | 0.290 |
| 5-HTTLPR: L/S vs S/S | 0.47 | 0.23 – 0.96 | **0.037** |
| Stressors$\times$ 5-HTTLPR: L/L vs S | 1.02 | 0.98 – 1.06 | 0.346 |
| Stressors$\times$ 5-HTTLPR: L/S vs S/S | 1.05 | 1.00 – 1.10 | 0.053 |
| *Note.* 1,483 observations. The standard deviations of random intercepts was 1.80 (person intercepts) and $\sigma_{u_{10k}}$ = 0.03 (person slopes). This table was created with the *tab_model* function of the R-package *sjPlot*.  *p*-values ≤ .05 are shown in boldface. | | | |
