# Supplementary material for: Highs and lows: Genetic susceptibility to daily events
Source: PLoS One. 2020 Aug 13;15(8):e0237001. doi: 10.1371/journal.pone.0237001 (PMC7425846; doi:10.1371/journal.pone.0237001)
Supplement: Attachment — Submitted filename: Response to Reviewers.docx [file pone.0237001.s008.docx]

Reviewer #1:

Comment 1: There is abundance of literature showing that personality traits are related to differential susceptibility. No matter what the authors mean with “differential susceptibility” if they mean variability in mood (states) or susceptibility to psychopathology. Neuroticism is defined a s a trait related to the predisposition to be “moody” and there is clear proneness for anxiety or affective disorders (e.g. De Moor et al., 2015).

Response:

We do not share the Reviewer’s assessment of an abundant literature linking neuroticism to differential susceptibility. „Susceptibility to psychopathology“ reflects the concept of vulnerability, which is distinct from differential susceptibility. There is a sound theoretical basis for the differential susceptibility concept (Belsky, 1997; Belsky et al., 2007; Pluess, 2015), and this theory represents the basis of our study. As outlined in the introduction of our paper, the concept of differential susceptibility was developed to show there are different patterns of reactivity to different environmental circumstances, which go beyond the concept of vulnerability. Differential susceptibility addresses predisposing factors which increase the susceptibility to both negative *and* positive events. While neuroticism surely is positively related to the vulnerability to negative events (Ormel et al., 2013; Shackman et al., 2016), we are not aware of compelling evidence showing that neuroticism also leads individuals to profit more from positive events, which is -the core of differential susceptibility theory. For example, individuals high in neuroticism show lower increases of positive affect in experimental inductions of positive mood (Larsen & Ketelaar, 1991), which is in agreement with Gray’s (1981) neuroticism model. Specifically in the EMA context, the negative affect of individuals high in neuroticism is more sensitive to negative, but not positive events (for a well-powered example see Longua et al., 2009).

Belsky, J. (1997). Theory Testing, Effect-Size Evaluation, and Differential Susceptibility to Rearing Influence: The Case of Mothering and Attachment. *Child Development*, *68*(4), 598. https://doi.org/10.2307/1132110

Belsky, J., Bakermans-Kranenburg, M. J., & van IJzendoorn, M. H. (2007). For better and for worse: differential susceptibility to environmental influences. *Current Directions in Psychological Science*, *16*(6), 300–304. https://doi.org/10.1111/j.1467-8721.2007.00525.x

Gray, J. A. (1981). A critique of Eysenck's theory of personality. In H. J. Eysenck (Ed.), A model for personality (pp. 246-276). New York: Springer.

Larsen, R. J., & Ketelaar, T. (1991). Personality and Susceptibility to Positive and Negative Emotional States. *Journal of Personality and Social Psychology*, *61*(1), 132–140. https://doi.org/10.1037/0022-3514.61.1.132

Longua, J., DeHart, T., Tennen, H., & Armeli, S. (2009). Personality moderates the interaction between positive and negative daily events predicting negative affect and stress. *Journal of Research in Personality*, *43*(4), 547–555. https://doi.org/10.1016/j.jrp.2009.02.006

Ormel, J., Jeronimus, B. F., Kotov, R., Riese, H., Bos, E. H., Hankin, B., Rosmalen, J. G. M., & Oldehinkel, A. J. (2013). Neuroticism and common mental disorders: Meaning and utility of a complex relationship. *Clinical Psychology Review*, *33*(5), 686–697. https://doi.org/10.1016/j.cpr.2013.04.003

Pluess, M. (2015). Individual Differences in Environmental Sensitivity. *Child Development Perspectives*, *9*(3), 138–143. https://doi.org/10.1111/cdep.12120

Shackman, A. J., Tromp, D. P. M., Stockbridge, M. D., Kaplan, C. M., Tillman, R. M., & Fox, A. S. (2016). Dispositional negativity: An integrative psychological and neurobiological perspective. *Psychological Bulletin*, *142*(12), 1275–1314. https://doi.org/10.1037/bul0000073

Comment 2: Bad argument, sorry. You are not aiming to demonstrate an association between 5-HTTLPR and personality. You aim to explain variance in susceptibility. If predictors are uncorrelated this is a clear advantage for explaining additional variance in your dependent variable.

(referring to our previous response: “A meta-analysis of large-scale genome-wide association studies reported no association between big five personality traits and serotonergic genes, including the 5-HTTLPR (Lot et al., 2017). Therefore, we think there is currently no sufficient basis to include personality measures in a paper that focuses on the 5-HTTLPR.)”

Response:

We assumed the reviewer was suggesting that neuroticism might mediate the effect of 5-HTTLPR on differential susceptibility. We agree, as the reviewer was not suggesting this relationship, our argument of no association between serotonergic genes and personality does not apply.

The aim of our study was to test the hypothesis that specifically the 5-HTTLPR is related to individual differences in differential susceptibility. As noted in the introduction and discussion, this hypothesis is embedded in the larger theory of the role of the serotonin system. It was not the aim of our study to find predictors which explain large amounts of variance in differential susceptibility. Single polymorphisms are known to explain only small amounts of variance, which are not practically meaningful for prediction, further highlighting the inconsistency between these two aims. We added the following phrase in response to a comment of the previous round of reviews, which makes this explicit:

“Last, although the effect sizes were reasonable for genetic studies on single polymorphisms, they are not large in absolute terms. Nevertheless, the main purpose of the present study was to test the theory that individual variability in the serotonin system is related to differential susceptibility”

Taken together, we still believe there is an insufficient basis to include a predictor of no interest (i.e. neuroticism) which is empirically unrelated to our predictor of interest (i.e. 5-HTTLPR) and has no clear straight-forward relationship to the outcome of interest (i.e. differential susceptibility; see response to comment 1).

Comment 3:
As I already said, the triallelic approach is common standard when genotyping for 5-HTTLPR. Technically, you can run this in the same analysis, with the exception with an enzymatic digestion in-between. I am confident that the laboratories will open soon. And one person alone can conduct the analyses safely alone in the lab.

Response: Unfortunately, we are currently still in no position to repeat those analyses. We added the following paragraph to the discussion:

„A further limitation is that we did not genotype^1^ for rs25531 A/G SNP within the 5-HTTLPR repetitive element, which has been reported to render the G allele within the l allele transcriptionally less efﬁcient (Wendland 2006), a finding that has not been consistently replicated (Martin 2007)”

^1^Footnote: Reanalysis of the available DNA samples was not possible due to Corona-associated lab closure.

Reviewer #2: I think the authors did a great job responding to my comments.
I only have two additional comments, which the authors may consider:

Comment 1.1: Regarding (a priori) power analysis (or not)/ comment 10: I my first review, I misread the relevant section, and I think this was for a good reason (or logic). If there were prior genetic studies on the same effect (Gunthert et al), then why not using those data for an a priori power analysis - just as the authors seem to have done post hoc? That would seem possible to me, and that's why I mistakenly thought an a priori power analysis has been done.

Response:

First, we changed the following sentence to clarify that we did not conduct an a priori power analysis before data collection to determine the sample size (changes in italics): “As such data was not available, *our* sample size was based *on the sample size* of other genetic EMA studies on the same effect (e.g. [33]).”

We also started a new paragraph after this sentence, to highlight the difference between sample size decision and post hoc power analysis.

For power estimation in linear mixed models, information are necessary on both (a) the expected effect size and (b) the expected covariance structure. While we were able to calculate a standardized effect size from Gunthert et al., the extraction of the covariance structure would not have been possible with the information given in the paper and would likely not be meaningful due to differences in the sampling plan. We tried to express this with the following sentence:

“To estimate the appropriate sample size for effect detection in EMA studies, prior data with a similar design are necessary [39]. As such data was not available, sample size was based on other genetic EMA studies on the same effect (e.g. [33])” [method section]

Therefore, we combined the previous effect size by Gunthert et al. with the empirical covariance structure of our design. We tried to express this more specifically in the results section (which we refer to in the method section on power for further detail):

“we tested the statistical power of our design based on our empirical covariance structure and the effect sizes reported by Gunthert et al. [33]. […] We followed the procedure described in Green and Macleod [49]” [results section]

Notably, in hindsight, it would have been possible to simulate a range of plausible true covariance structures and determine a power range before data collection. Unfortunately, we did not consider this approach at the time of study planning.

Comment 1.2: Besides, if not for using information of a prior study for a power analysis, I don't see how a prior study can inform about what sample size to aim for.

Response:

We wrote: “A simulated post-hoc power analysis based on our empirical data indicated a power of 61.70% to detect a previously reported effect size [33]”

The term “post-hoc power analysis”, as we are familiar with it, refers to the estimation of power, given that all other relevant parameters are fixed (i.e. sample size, alpha level, effect size, and covariance structure) and therefore does not provide a means of sample size estimation, but rather a means to indicate what the power of a given study has to detect an effect size of interest.

This is substantially inferior to an a priori power analysis for sample size estimation, but we included it nevertheless, as prior reviewers consistently expressed the need to give some indication of achieved statistical power for reasonable effect sizes, due to the ongoing discussion concerning limited power in candidate gene studies.


2. (referring to my comment 22): Regarding this and some of my other comments on the discussion, the authors seem to have a slightly different opinion, in some instances, on where and how to set the emphasis, which is fair enough and totally ok for me. The one minor point where I think it is worth giving it another thought is their reference to investigate reactivity as a dynamic system. I totally agree about the importance of testing such effects on the within-subject level. But I'm not sure it is clear what they investigated as a dynamic system. Reactivity is dynamic per definition, so this sentence seems a little tautological.

If we are correct, the phrase the reviewer points towards is in the introduction (we hope we didn’t overlook a similar phrase in the discussion): “However, both differential susceptibility and the vulnerability to stressors are the property of a dynamic system that only manifests over time”.

We completely agree that reactivity is dynamic per definition. This is what we try to express with this sentence. Unfortunately, most studies on differential susceptibility relied on cross-sectional between-person correlations to still draw conclusions for reactivity. We tried to highlight this in the phrase before the dynamic system reference: “Inconsistent findings in this area might be partly due to the fact that the overwhelming majority of studies attained cross-sectional data, and correlations were usually computed on a between-person level (e.g. [18])”

Thus, while we ourselves entirely agree with you and find this statement to be self-evident, we included it because we are under the impression this is not clear to most parts of the literature which our study is based on.

If the reviewer finds this explanation insufficient, we are of course open for further comments and suggestions.
