# Supplementary material for: Highs and lows: Genetic susceptibility to daily events
Source: PLoS One. 2020 Aug 13;15(8):e0237001. doi: 10.1371/journal.pone.0237001 (PMC7425846; doi:10.1371/journal.pone.0237001)
Supplement: Attachment — Submitted filename: ResponseToReviewers.rtf [file pone.0237001.s009.rtf]

Reviewer #3: I was asked to review this manuscript, while not being involved in the previous revisions. I honestly have to say I started out skeptical, due to the current state of the field with regard to candidate gene studies. However, I was pleasantly surprised. I think the authors did a great job in rigorously testing the hypotheses, and were very nuanced and complete in interpreting their findings. This included discussing the limitations, but also the controversies surrounding candidate gene studies.

I was a bit hesitant with regard to the difference scores that were used to test differential susceptibility. I think these scores have many problems, as the authors also state in their Discussion. The analyses split for events and affect provide much more insight into the mechanisms. I am also not convinced that averaging the event scores is the best solution, as certain events may be more impactful than others. However, given the state of the manuscript and potential issues with multiple testing, I will not ask for different analyses.
Response:
We thank the Reviewer for her critical and at the same time validating comments concerning our manuscript. We will respond to the individual comments below:


Comment 1
the authors use a three-level model (moment, day, person); did they check whether the second (day) level was required? I could not find whether the study period included week days only or also weekend days.
Response:
We added the following information to the method section: 

“Assessments always started on a Thursday to sample both working days and weekend days in a similar frequency”
“A likelihood ratio test for an intercept-only model with mood as the dependent variable indicated that including the day level increased model fit significantly (÷²(1) = 55.34, p < .001; 46.6% variance between participants, 11.7% between days, and 47.7% between measurement occasions)”


Comment 2
I agree with reviewer 1 that the tri-allelic approach is preferable, so if it would be possible, this would be nice to add. However, I can also imagine that labs are still not working on full speed currently.

Response: 
There are two issues regarding genotyping of rs25531. First, as the reviewer pointed out, our University is still running on a limited scale and our lab is currently allowed to work with 50% capacity only. There is still a major backlog of time-critically funded studies. Therefore, the re-analysis of this sample can unfortunately not be accomplished in the foreseeable future.
The second reason are doubts surrounding the effects of rs25531 on 5HTT expression. In 2007, Martin et al. comprehensively analyzed 55 SNPs in 100 kb surrounding the SLC6A4 gene regarding their effect on gene expression. In addition to the VNTR („5HTTLPR“), two other SNPs (both with low minor allele frequency) had a significant effect, but not rs25531. 


Figure from Martin et al. (2007). Mapping regulatory variants for the serotonin transporter gene based on allelic expression imbalance. Mol Psychiatry.

We hope these reasons, as well as their acknowledgment in the limitations section, are acceptable.

Comment 3:
Finally, I would like to stress that I think that pre-registration and direct replication is essential in these studies, and I would advise the authors to do this next time. Particularly in this field, pre-registration may help to solve the inconsistencies in previous work.
Response:
We entirely agree with the reviewers' comment and are continuously working to improve our reproducibility and replicability practices.
